# The Effect of Antipsychotics on Prolactinoma Growth: A Radiological and Serological Analysis

**DOI:** 10.7759/cureus.49342

**Published:** 2023-11-24

**Authors:** Umar S Durrani, Satvik Vasireddy, Maha Z Arshad, Awais Paracha, Maria A Paracha, Fatima Waheed, Ali Abid, Zohair Siddiqui, Michael Thomure

**Affiliations:** 1 Medicine, Saint Louis University School of Medicine, Saint Louis, USA; 2 Radiology, Touro University Nevada College of Osteopathic Medicine, Saint Louis, USA; 3 School of Medicine, Saint Louis University School of Medicine, Saint Louis, USA; 4 Oncology, Saint Louis University School of Medicine, St. Louis, USA; 5 College of Arts and Sciences, University of Virginia, Charlottesville, USA; 6 Oncology, New York Institute of Technology College of Osteopathic Medicine, Old Westbury, USA; 7 College of Arts and Sciences, Saint Louis University, St. Louis, USA; 8 Internal Medicine, Saint Louis University School of Medicine, Saint Louis, USA; 9 Obstetrics, Gynecology, and Women’s Health-Repo Endocrinology, Saint Louis University School of Medicine, Saint Louis, USA

**Keywords:** antipsychotic medication effect on prolactinoma size, prolactinoma growth, antipsychotic medication, psychiatric disorders neurologic diseases, prolactinoma

## Abstract

Many antipsychotic (AP) medications work by reducing dopamine levels. As hyperdopaminergia is known to cause psychosis, antipsychotics work to relieve these symptoms by antagonizing dopamine receptors and lowering dopamine levels. Dopamine is also a known negative modulator of the prolactin pathway, which allows for drug agents like dopamine agonists (DAs) to be incredibly effective in managing tumors that secrete excess prolactin (prolactinomas). While the effects of DAs on prolactinoma size and growth have been studied for decades, the effects of APs on prolactinoma size remain to be seen. We hope to investigate the effects of APs on prolactinomas by conducting a thorough PubMed search, including patients with diagnosed prolactinoma on concurrent AP therapy. Our search led to 27 studies with a total of 32 patients. We identified themes regarding seven antipsychotics: risperidone, haloperidol, amisulpride, thioridazine, aripiprazole, olanzapine, and clozapine. Risperidone, haloperidol, amisulpride, and thioridazine caused a significant increase in prolactin in most cases where they were used, and prolactin decreased after their discontinuation. For example, risperidone discontinuation resulted in a decrease in prolactin levels by an average of 66%, while haloperidol, amisulpride, and thioridazine discontinuation lowered prolactin by an average of 82%, 72%, and 89.7%, respectively. However, there were some exceptions in regard to risperidone, haloperidol, and thioridazine, where prolactin levels were not as severely affected. Aripiprazole, olanzapine, and clozapine all had significant reductions in prolactin levels when patients were switched from another antipsychotic, such as risperidone or haloperidol. The average percent decrease in prolactin when switched to aripiprazole was 67.65%, while it was 54.16% and 68% for olanzapine and clozapine, respectively. The effect of individual antipsychotics on prolactinoma size was difficult to ascertain, as imaging was not obtained (or indicated) after every antipsychotic switch, and many patients were taking dopamine agonists concurrently. Therefore, it would be difficult to ascertain which factor affected size more. Also, some patients received surgery or radiotherapy, which completely negated our ability to make any assertions about the effects of certain pharmacological agents. Although it is difficult to ascertain the role that antipsychotic medications play in the formation of prolactinoma, we have found that the cessation of certain antipsychotic medications may lead to a reduction in prolactin levels and possibly the presence of a measurable prolactinoma.

## Introduction and background

Prolactinomas are the most common tumors of the pituitary gland and result in excess secretion of the hormone prolactin. This tumor can cause infertility, growth and development defects, osteoporosis, and other pituitary gland hormone deficiencies. Common symptoms include excessive lactation, hair growth abnormalities, and decreased muscle size. MRI is the most favored imaging method for this tumor [1]. Decreasing or stable prolactin levels have also been shown to be effective surrogate markers for prolactinoma tumor shrinkage or stabilization [2].

Dopamine is an important neurotransmitter involved in the regulation of prolactin. It does this by binding to receptors on the prolactin-secreting lactotrophs, thus inhibiting prolactin release [3].

Dopamine agonists (DAs) such as cabergoline and bromocriptine are commonly used as treatments for prolactinomas (though cabergoline is favored for the treatment of prolactinoma and idiopathic hyperprolactinemia) [4]. Dopamine is also implicated in the pathogenesis of various psychiatric disorders. Excessive activity of this neurotransmitter is known to contribute to the symptoms of schizophrenia [5]. Therefore, the key mechanism of action by which antipsychotics (APs) address many psychiatric diseases is by decreasing dopamine levels. Our knowledge of the relationship between dopamine levels and prolactin regulation raises the question of the effect of antipsychotics on prolactinoma growth via dopamine antagonism. With almost 2% of the total U.S. adult population taking antipsychotics, it is essential to determine if antipsychotic therapies play a role in prolactinoma prognosis [6].

## Review

Methods

This work was previously presented at the Endocrine Society 2023 conference.

Literature Search

This review was performed in accordance with the Preferred Reporting Items for Systematic Review and Meta-Analysis Protocols (PRISMA-P) [7]. Beginning March 7, 2023, two research team members (U.D. and A.A.) reviewed articles on the PubMed/MEDLINE database. Search queries were not limited to a specific time frame. However, filters were applied to the search engine for the English language and human species. Search queries were further sequenced to correspond with primary and secondary search terms. Primary search terms correlated to prolactinoma are: prolactinoma, prolactinoma treatment, and prolactinoma growth. Secondary search terms referred to the various terminology associated with antipsychotics: antipsychotics, antipsychotic medications, schizophrenia, schizoaffective disorder, psychiatric disorder, psychiatric conditions, major depressive disorder with psychotic features, psychosis, and bipolar disorder.

Study Selection

**Figure 1:**
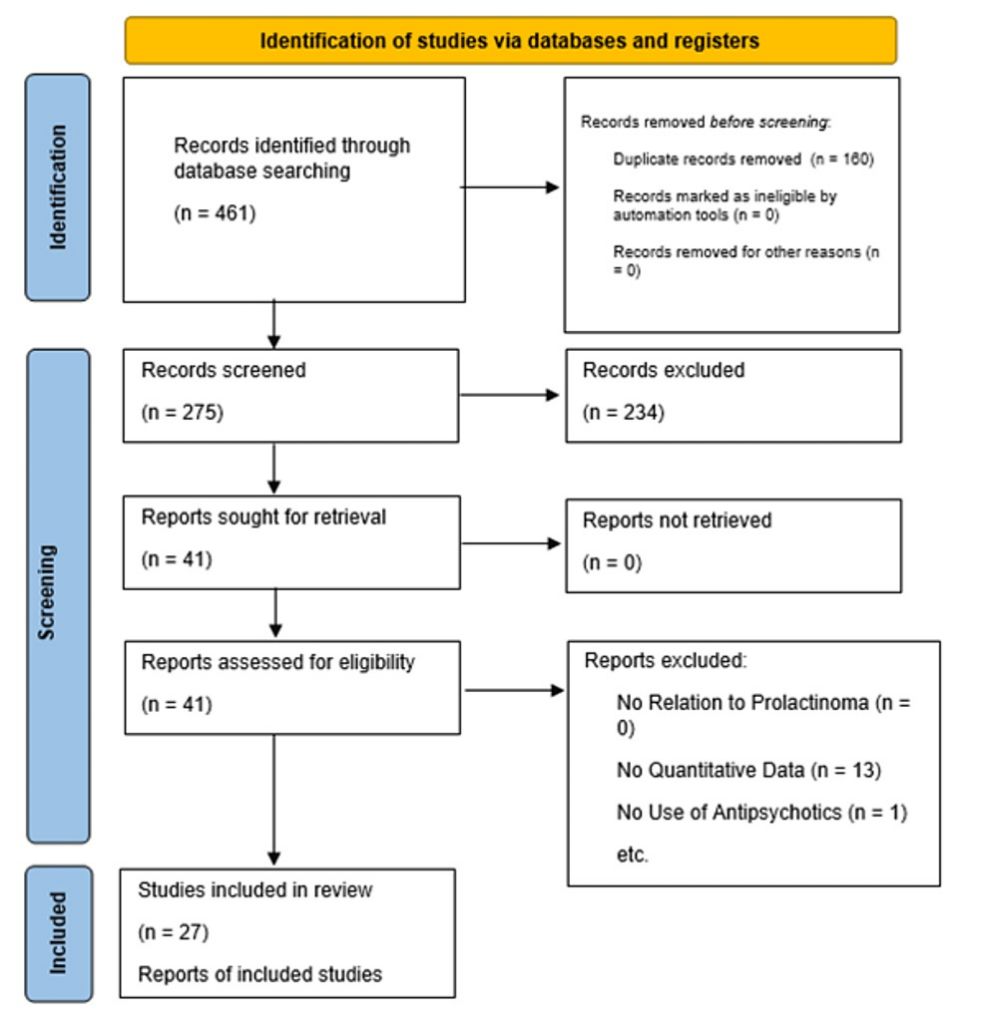
PRISMA flowchart

Based on the study inclusion and exclusion criteria, three reviewers (U.D., A.A., and A.P.) independently assessed the eligibility of the relevant papers. Exclusion criteria did not include treatment intervention or outcome in order to conserve a wide range of studies. The relevant studies consisted of any paper that discussed antipsychotic medications in the context of prolactinoma growth. Furthermore, studies were excluded if patients did not report both prolactinoma diagnosis and usage of antipsychotics, had inadequate methodologies, or were duplicate studies. To combat the risk of individual bias, both reviewers independently assessed all selected studies using a team-based approach to determine inclusion or exclusion. Figure 1 displays a PRISMA flow diagram of the systematic process for study selection.

Data Extraction and Analysis

The following data were collected from each included study: psychiatric diagnosis, dopamine agonists, antipsychotics used, changes in prolactin, MRI changes, radiation therapy, and other significant treatment details, if applicable. In addition, qualitative data from each study sample (i.e., brain imaging and serum prolactin) was used as a surrogate marker to evaluate prolactinoma growth. Table 1 outlines the individual patient data and treatment course.

**Table 1:** Patient descriptions

Patients	Psychiatric diagnoses	Dopamine agonist	Antipsychotics used	Changes in prolactin	MRI changes	Surgery (Y or N)	Radiation (Y or N)	Significant treatment details
Bamarinejad et al. [8]	Schizophrenia	Cabergoline	Risperidone perphenazine aripiprazole clonazepam quetiapine	1st: 1500 ng/mL; 2nd: 52 ng/mL (96.5% decrease)	Not reported	Y	N	The patient was a 29-year-old male with an 8-year history of psychiatric disorders and was ultimately diagnosed with schizophrenia. The patient had been on Risperidone and Perphanize, which were ultimately discontinued when the macroprolactinoma diagnosis was confirmed. These were later replaced by Aripiprazole and Clonazepam, and the patient experienced an improvement in visual symptoms, but psychiatric symptoms still remained. The dosages of each agent were unspecified. Ultimately, the patient had transsphenoidal surgery and the patient was started on quetiapine, in addition to the cabergoline and levothyroxine prescribed previously.
Ali et al. [9]	Paranoid schizophrenia	N/A	Olanzapine, risperidone	1st: 1986.5 ng/mL; 2nd: 291.8 ng/mL; (85.3% decrease); 3rd: 314.5 ng/mL; (7.78% increase post-risperidone)	Initial size: 1.6 cm; final size: no change (5-years post-treatment)	Y	N	The patient was a 25-year-old male with paranoid schizophrenia who had successful treatment with olanzapine (10 mg/day). The patient received a first-episode psychosis MRI, which revealed a pituitary macroadenoma measuring 1.6 cm in size and a prolactin level of 1986.5 ng/ml. The patient was subsequently operated on, and prolactin levels fell. The patient was put on long-acting risperidone (dosage unspecified) and has been psychologically well since, with a small increase in overall prolactin levels.
Freeman et al. [10]	Schizophrenia	Cabergoline bromocriptine	Risperidone, olanzapine, ziprasidone, aripiprazole	1st: 171 ng/mL; 2nd: 3.29 ng/mL (98.1% decrease); 3rd: 4.79 ng/mL (45.6% increase)	Not reported	N	N	The patient was a 23-year-old female who had a 4-year history of schizophrenia and risperidone, olanzapine, and ziprasidone use, as well as image-documented macroadenoma following risperidone cessation. Dosages of agents were unspecified. The patient was prescribed both cabergoline and bromocriptine (unknown doses), however, neither the hyperprolactinemia nor psychiatric symptoms were well-controlled. The patient was eventually taken off cabergoline, and given IM Lorazepam and Ziprasidone, which was followed by a continuous prescription of oral aripiprazole (30 mg/day).
Akkaya [11] (1st Patient Reported)	Schizophrenia	N/A	Amisulpride, biperiden	1st: 21.7 ng/mL; 2nd: 109.10 ng/mL (2 weeks); (402.8% increase); 3rd: 124 ng/mL (3 months); (13.6% increase); 4th: 106.2 ng/mL (6 months); (14.4% decrease)	Initial size: not reported; final size: 5 mm, with two foci measuring 2 and 3 mm suggestive of microadenomas.	N	N	The patient was a 55-year-old man with a 26-year history of schizophrenia and had previously been prescribed haloperidol and biperiden before. However, the patient reported no antipsychotic use for 7 years prior to the study. Amisulpride was given at 200 mg/day and increased to 800 mg/day after 14 days.
Akkaya [11] (2nd Patient Reported)	Schizophrenia	N/A	Amisulpride	1st: 18.55 ng/mL; 2nd: 65.8 ng/mL (2 weeks); (254.7% increase); 3rd: 72.9 ng/mL (5 months); (10.8% increase); 4th: 60.28 ng/mL (6 months); (17.1% decrease)	Initial size: not reported; final size: not reported; however, there was a 2 mm foci suggestive of a microadenoma	N	N	The patient was a 36-year-old man with a 9-year history of schizophrenia and had previously been prescribed risperidone, olanzapine, fluvoxamine, valproic acid, and biperiden before. However, the patient reported no antipsychotic use for 6 years prior to the study. Amisulpride was given at 200 mg/day and increased to 800 mg/day after 14 days.
Akkaya [11] (3rd Patient Reported)	Schizophrenia	N/A	Amisulpride, biperiden, aripiprazole	1st: 26.7 ng/mL; 2nd: 367.0 ng/mL (1274.5% increase); 3rd: 424.0 ng/mL (4 months); (15.53% increase); 4th: 285.0 ng/mL (6 months) (32.8% decrease); 5th: 17.78 ng/mL (93.76% decrease)	Initial size: not reported; final size: not reported; however, there was a 2 mm microadenoma	N	N	The patient was a 28-year-old woman with a 4-year history of schizophrenia and had previously been prescribed olanzapine, thioridazine, alprazolam, quetiapine, and risperidone before. The patient had a history of poor compliance. However, the patient reported no antipsychotic use for 20 days prior to the study. Amisulpride was given at 200 mg/day and increased to 800 mg/day after 14 days. The patient was switched to aripiprazole, and after 2 months of treatment, prolactin levels decreased to 17.78 ng/mL.
Rad et al. [12]	Bipolar disorder	None	Risperidone, haloperidol, olanzapine	1st: 63.2 ng/mL; 2nd: 55–85 ng/mL (10.8% increase); 3rd: 17 ng/mL (75.7% decrease)	Initial size: 11/8 mm; final size: “normal”	N	N	The patient was a 13-year-old who was prescribed 2.25 mg/day of risperidone for symptoms aligning with an acute psychotic episode. After two months of treatment, the patient had a prolactin level of 63.2 ng/mL. Therefore, the dose of risperidone was decreased to 1 mg/day. Prolactin level still ranged from 55–85 ng/mL. Continued treatment with risperidone revealed a pituitary mass of 11/8 mm at 10 months. Therefore, risperidone was discontinued. Later, the patient was started on haloperidol, which was ultimately switched to 2.5 mg/day of olanzapine. The patient’s last recorded prolactin level was 17 ng/mL.
Mendhekar et al. [13]	Bipolar disorder	Bromocriptine	Risperidone, olanzapine, lithium carbonate	1st: 124.8 ng/mL; 2nd: 56 ng/mL (55.1% decrease); 3rd: 36 ng/mL (35.7% decrease); 4th: 42 ng/mL (16.7% increase); 5th: 18 ng/mL (57.1% decrease); 6th: 12 ng/mL (33.3% decrease)	Initial size: 3-4 mm hypodensities in the pituitary gland; final size: normal	N	N	The patient was a 35-year-old woman who was being managed with 900 mg/day of lithium and 6 mg/day of risperidone. Due to complaints of amenorrhea and galactorrhea, risperidone was discontinued completely and lithium was maintained as a monotherapy. Imaging demonstrated 3-4 mm hypodensities in the pituitary gland and serum studies showed a prolactin level of 124.8 ng/mL. Risperidone cessation led to a decrease in prolactin levels to 56 ng/mL. 2.5 mg/day of olanzapine was added to the lithium therapy. Prolactin decreased to 36 ng/mL and 42 ng/mL 12.5 mg/day of bromocriptine was added, and this decreased prolactin to 18 ng/mL and 12 ng/mL.
Arcari et al. [14]	Schizoaffective disorder	N/A	Thioridazine, ziprasidone	1st: 83.8 μg/L; 2nd: 100.1 μg/L (19.5% increase); 3rd: 41.7 μg/L (58.3% decrease); 4th: 11.7 μg/L (71.9% decrease); 5th: 13.1 μg/L (12.0% increase); 6th: 11.1 μg/L (15.3% decrease); 7th: 7.1 μg/L (36.0% decrease); 8th: 12.0 μg/L (69.0% increase); 9th: 11.6 μg/L (3.33% decrease); 10th: 7.0 μg/L (39.7% decrease); 11th: 10.2 μg/L (45.7% increase); 12th: 11.7 μg/L (14.7% increase)	Initial size: 2 mm; final size: normal morphology	N	N	The patient was a 36-year-old woman who received electroconvulsive therapy and 50 mg/day of thioridazine in the past but was switched to 2 mg/day of risperidone. After some time of risperidone therapy, prolactin was discovered to be 83.8 μg/L. Subsequent imaging revealed a pituitary mass measuring 2 mm. Prolactin continued to increase up to 100.1 μg/L. Therefore, risperidone was discontinued and 40 mg/day of ziprasidone was started. The dosage of ziprasidone was slowly increased by 40 mg every other week, while risperidone dosage was decreased by 0.5 mg in the same time frame. Eventually, the patient was only on 160 mg/day of ziprasidone, which conferred a decrease in prolactin down to 41.7 μg/L, Subsequent measurements were even lower, fluctuating around the 7-13 range.
Broekhof et al. [15]	Chronic psychotic disorder	Quinagolide	Risperidone, aripiprazole	1st: 470 μg/L; 2nd: 210 μg/L (55.3% decrease); 3rd: 123 μg/L (41.4% decrease)	Initial size: not specified; final size: “decreased”	Y	N	The patient was a 53-year-old woman with chronic psychiatric issues and some mental retardation that was treated with 1 mg/day for 15 years. Prolactin levels were found to be 470 μg/L, and a pituitary macroadenoma was found via imaging. It was subsequently transected via transsphenoidal microsurgery. Quinagolide was started at 75 μg/day, and risperidone was switched for aripiprazole at 15 mg/day but was increased to 30 mg soon after. Prolactin levels decreased down to 123 μg/L after these changes.
Gupta et al. (1st Patient Reported) [16]	Acute psychosis/potential bipolar disorder	Cabergoline	Risperidone, valproate	1st: 401 ng/mL; 2nd: 10 ng/mL (97.5% decrease); 3rd: 201 ng/mL (1910% increase); 4th: 12 ng/mL (94.0% decrease); 5th: 15 ng/mL (25% increase)	Initial size: 5 mm × 6 mm; final size: unchanged	N	N	The patient was a 34-year-old woman who presented with galactorrhea and amenorrhea; prolactin was discovered to be 401 ng/mL alongside a pituitary microadenoma measuring 5 mm × 6 mm. Cabergoline was started at 0.5 mg/week, which decreased the prolactin to 10 ng/mL. After 8 months, the patient began exhibiting psychiatric symptoms, so 2–6 mg/day of risperidone, as well as 100 mg/day of sertraline. While psychiatric symptoms were controlled, prolactin levels increased. The patient discontinued both cabergoline, risperidone, and sertraline, and was placed on valproate at a dosage of 750 mg/day. This led to a stabilization of mood symptoms and a lowering in prolactin levels to 12 ng/mL. Eventually, all medications were slowly tapered and prolactin stabilized at 15 ng/mL.
Gupta et al. (2nd patient-reported) [16]	Acute psychosis	Cabergoline	Olanzapine, aripiprazole	1st: 169 ng/mL; 2nd: 9 ng/mL (94.7% decrease)	Initial size: 2 mm × 3 mm; final size: normal	N	N	The patient was a 29-year-old woman who presented with amenorrhea, hirsutism, and headache. Prolactin was revealed to be 169 ng/mL and a pituitary microadenoma of 2 mm × 3mm. Cabergoline was stated at 0.5 mg/week, which improved prolactin levels, but induced psychosis. The patient was given lorazepam once and escitalopram for depression-like symptoms. However, it was eventually switched to olanzapine at a dosage of 10 mg/day, though no psychiatric improvement was seen. Cabergoline and olanzapine were both discontinued, which helped resolve most symptoms after 3 weeks. 5 mg/day of aripiprazole was started, but the patient developed akathisia, so it was discontinued as well. Prolactin had decreased to 9 ng/mL by this point.
Sheldrick et. al [17]	Acute psychosis	Bromocriptine	Risperidone, aripiprazole	1st: 39,160 mU/L; 2nd: 9400 mU /L (76.0% decrease); 3rd: 159 mU /L (98.3% decrease)	Initial scan reported a pituitary adenoma of unspecified size; final scan showed no progression of adenoma	N	N	The patient was a 39-year-old woman who presented with psychotic symptoms and a reported prolactinoma for which she took 2.5 mg/day of bromocriptine. The patient was non-compliant for 2 months prior to the episode. The patient was given risperidone for a short time but was quickly switched to aripiprazole due to contraindication in a patient with prolactinoma. 15 mg/day of aripiprazole and 2.5 mg/day of bromocriptine led to a decrease in prolactin levels and normalization of psychiatric symptoms.
Pal et al. [18]	Acute psychosis	Bromocriptine	Valproate, risperidone, haloperidol	1st: 260.8 ng/mL; 2nd: 45 ng/mL (82.7% decrease); 3rd: 35 ng/mL (22.2% decrease)	Initial size: 10–11 mm; final size: 12 mm	N	N	The patient was a 19-year-old who was hospitalized for a psychiatric episode, during which it was discovered that the prolactin level was 260.8 ng/mL. The patient had been treated with unspecified doses of haloperidol, lorazepam, and benztropine, but later was switched to 2 mg/day of risperidone and 250 mg/day of valproate (Depakote). These were both discontinued (gradually), and the patient was started on 5 mg/day of olanzapine and 600 mg/day of carbamazepine. This switch led to prolactin decreasing to 35 ng/mL. The patient was ultimately managed with 5 mg/day of olanzapine, 600 mg/day of carbamazepine, 1 mg/day of benztropine, and 15/mg per day of bromocriptine.
Koves et al. (2nd Patient Reported) [19]		Cabergoline	Risperidone	1st: 137 μg/L; 2nd: 8 μg/L (94.2% decrease)	Initial size: 10 mm × 12 mm × 15 mm; final size: no change	N	N	The patient was a 16-year-old girl with intellectual disabilities and endocrine symptoms such as dysmenorrhea and galactorrhea. The patient also reported headaches, and this all occurred ~6 months after the start of a risperidone prescription (3 mg/day). Serum studies and imaging showed a prolactin level of 137 μg/L, and imaging showed a pituitary mass measuring 10 mm × 12 mm × 15 mm. The patient was started on 1.5 mg/week of cabergoline, with dexamphetamine tapered off, and 1 mg/day of risperidone. Prolactin decreased to 8 μg/L on this regimen.
Koves et al. (3rd Patient Reported) [19]	Bipolar disorder	N/A	Risperidone	1st: 135 μg/L; 2nd: 76 μg/L (43.7% decrease); 3rd: 30 μg/L (60.5% decrease)	Initial scan: 13.7 mm × 7 mm × 7 mm; no other scan obtained	N	N	The patient was a 16-year-old girl who presented with 6 months of psychiatric symptoms and was subsequently placed on increased doses of risperidone. These went up to 6 mg/day, which was used for 10 days before symptoms of prolactinoma became apparent. Risperidone was discontinued and prolactin fell from 135 μg/L ultimately down to 30 μg/L.
Perroud et al. [20]	Unspecified psychotic disorder		Amisulpride, quetiapine, risperidone, haloperidol, olanzapine	1st: 101 ng/mL; 2nd: 22.4 ng/mL (77.8% decrease); 3rd: 87.5 ng/mL (290.6% increase); 4th: 48.6 ng/mL (44.5% decrease); 5th: 11 ng/mL (77.4% decrease)	Initial size: 5 mm; final size: not reported	N	N	The patient was a 38-year-old woman with recurrent MDD who abruptly stopped taking Biperiden, and was considered to be in withdrawal. The patient was placed on 300 mg/day of amisulpride, which improved psychiatric symptoms, but prolactin levels were discovered to be 101 ng/mL. Imaging revealed a pituitary mass measuring 5 mm. Amisulpride was thus withdrawn, and quetiapine was started at 100 mg/day. This led to a decrease in prolactin to 22.4 ng/mL. The patient was then non-compliant with quetiapine due to side effects, and the patient was given 3 mg/day of risperidone at her next episode, which was switched to haloperidol (1 mg/day). Risperidone caused the prolactin levels to increase to 87.5 ng/mL. Haloperidol brought it down to 48.6 ng/mL. Olanzapine was started at 5 mg/day, and prolactin decreased to 11 ng/mL.
Liao et al. [21]	MDD with delusions	Cabergoline	Risperidone, aripiprazole	1st: 167 ng/mL; 2nd: 5.82 ng/mL (96.5% decrease)	Initial size: 2–3 mm; final size: not reported	N	N	The patient was a 48-year-old woman with a history of MDD who had been treated with paroxetine and risperidone in the past. The patient had another episode which was treated with 3 mg/day of risperidone and 20 mg/day of paroxetine, but the clinical response was poor, and prolactin was discovered to be 167 ng/mL. Imaging showed a 2-3 mm pituitary mass. Risperidone was switched to aripiprazole (5 mg/day), along with a long-acting dopamine agonist (Dostinex/cabergoline) at a dosage of 1 mg/day. Prolactin levels decreased to 5.82 ng/mL, and the patient was taken off aripiprazole, only continuing the long-acting cabergoline.
Andrade et al. [22]	Schizophrenia	Bromocriptine quetiapine	Haloperidol, biperiden	1st: 3616 ng/mL; 2nd: 286.7 ng/mL (post-op) (92.1% decrease); 3rd: 121 ng/mL (6 months post-op) (57.8% decrease); 4th: 56.3 ng/mL (post-radiation) (53.5% decrease)	Initial size: 25 mm × 20 mm × 12 mm; final size: not reported	Y	Y	The patient was a 39-year-old male who had been using haloperidol and biperiden for approximately 8 years. At age 37, the patient presented with bitemporal hemianopsia and a prolactin level of 3616 ng/mL. MRI showed a macroprolactinoma which measured 25 mm × 20 mm × 12 mm in size. Haloperidol (5 mg/day) and biperiden (2 mg/day) were discontinued, with subsequent surgery, alongside bromocriptine (2.5 to >5 mg/day) and quetiapine (200 to >400 mg/day) prescription. The patient also received radiation therapy to eliminate residual tumor tissue.
Konopelska et al. [23]	Schizophrenia	Bromocriptine	Haloperidol, fluphenazine decanoate, clozapine, lithium, perazindimalonat	1st: >1000 ng/mL; 2nd: ~500 ng/mL (50.0% decrease); 3rd: 130 ng/mL (74.0% decrease)	Initial size: 16 mm × 19 mm; final size: 8 mm	N	N	The patient was a 39-year-old woman with a 25-year history of schizophrenia, treated with a variety of medications such as diazepam, metofenazate, haloperidol (4.5 to >9 mg/day to >27 mg/day), biperiden (12 mg/day), fluphenazine decanoate (6.25 mg/week to > 50 mg/3 weeks), clozapine (150 mg/day), and lithium (900 mg/day). The patient presented with prolactin levels over 1000 ng/mL and a macroadenoma 16 mm × 19 mm in size. The patient has been prescribed bromocriptine (1.25 mg/day to >7.5 mg/day to > 18.75 mg/day), which was discontinued during psychiatric episodes but reinstated afterward. After 4 years, prolactin levels were finally able to be reduced to 500 ng/mL, and eventually, 130 ng/mL.
Daradkeh et al. [24]	Schizophrenia	Bromocriptine	Penfluridol, thioridazine, haloperidol	1st: 53 ng/mL; 2nd: 45 ng/mL (15.1% decrease); 3rd: 4.8 ng/mL (89.3% decrease)	Size not reported	Y	N	The patient was a 24-year-old with a long history of schizophrenia, with penfluridol (20 mg/twice weekly) and thioridazine (150 mg/day) prescriptions. Due to a suspected prolactinoma (imaging showed no abnormalities, but the patient had all symptoms), bromocriptine was started at 7.5 mg/day and later doubled to 15 mg/day. Penfluridol was discontinued, keeping the thioridazine and bromocriptine prescriptions active. Pituitary adenoma was finally discovered on the third round of imaging, and the patient underwent transsphenoidal resection of the tumor. Psychiatric symptoms returned while she was in the hospital and not taking thioridazine, so she was discharged on 15 mg/day of haloperidol and 6 mg/day of benzhexol, which has controlled all symptoms.
Bakker et al. [25]	Acute psychosis	Cabergoline quinagolide bromocriptine	Aripiprazole, haloperidol	1st: 2.25 IU/L; 2nd: 2.99 IU/L (32.9% increase); 3rd: 5.1 IU/L (70.6% increase); 4th: 0.63 IU/L (87.6% decrease); 5th: 1.58 IU/L (50.8% increase)	Initial size: 8 mm; 2nd scan: 6 mm; final size: 3 mm	N	N	The patient was a 30-year-old woman who presented with amenorrhea and headaches, with a history of psychiatric illness. She was initially placed on cabergoline and then quinagolide, which resolved symptoms, but they reappeared when the patient ceased taking the medication due to side effects. Prolactin level was discovered to be 2.25IU/L, alongside an 8 mm pituitary microadenoma. The patient was started on 2.5 mg/day of bromocriptine, but the patient began having psychiatric symptoms, and aripiprazole was started at 7.5 mg/day. This helped bring the prolactin level down to 0.63IU/L, but aripiprazole was discontinued later on due to pregnancy (the patient had a miscarriage on aripiprazole previously). Haloperidol was used in the patient, but the prolactin level increased to 1.58IU/L; therefore, she was switched back to aripiprazole 3.75 mg/day (initially with haloperidol, but it was tapered off).
Takeda et al. [26]	Delusions of dermatozoiasis	Bromocriptine	Haloperidol, thioridazine	1st: “high”; 2nd: “Normal” (see paper for chart)	Initial size: not reported; final size: not reported			The patient was a 68-year-old woman with visual symptoms and delusions of dermatozoiasis. The patient was trialed on multiple medications, including chlordiazepoxide (40 mg/day), diazepam (15 mg/day), haloperidol (15 mg/day), thioridazine (150 mg/day), chlorpromazine (200 mg/day), meclofenoxate (800 mg/day) and kallidinogenase (unspecified dose) to no effect. The patient was then put on bromocriptine and was given 7.5 mg/day, and PRL was normalized, but psychotic symptoms remained. Haloperidol was started at 3 mg/day, and the delusions disappeared.
Melkersson et al. (1st Patient Reported) [27]	Bipolar disorder	Bromocriptine, quinagolide	Haloperidol, lithium citrate	1st: 350 μg/L; 2nd: 223–251 μg/L (32.3% decrease); 3rd: 20 μg/L (91.6% decrease)	Initial size: 1 cm; final size not reported	Y	N	The patient was a 48-year-old man who had been on 2 mg/day of haloperidol and an unspecified dose of lithium citrate. Serum studies revealed a prolactin level of 350 μg/L and imaging studies showed an adenoma measuring 1 cm in diameter. Haloperidol was discontinued and bromocriptine was started at 2.5 mg/day. Surgery was attempted but was unsuccessful in removing the adenoma. Quinagolide (up to 0.375 mg/day) and 2 mg/day of haloperidol were used, and the prolactin level decreased to 20 μg/L.
Melkersson et al. (3rd Patient Reported) [27]	Paranoid schizophrenia	Bromocriptine	Remoxipride, haloperidol, zuclopenthixol, clozapine	1st: 2310 and 2630 μg/L; 2nd: 600 μg/L (75.7% decrease); 3rd: 130 μg/L (78.3% decrease); 4th: 400 μg/L (207.7% increase); 5th: 50 and 80 μg/L (83.8% decrease); 6th: 16–29 μg/L (65.4% decrease)	Initial size: 1 cm × 1.5 cm in size; final size: “decreased substantially”	Y	Y	The patient was a 23-year-old man who developed psychosis after prolonged treatment with bromocriptine. Remoxipride was started and titrated up to 300 mg/day. This, however, led to an increase in prolactin levels up to 400 μg/L, so remoxipride was discontinued, and bromocriptine was reinstated. Psychiatric symptoms returned, so remoxipride was restarted at 300 mg/day, and surgery was attempted to remove the pituitary tumor, but it was aborted due to profuse bleeding. Radiation therapy was applied, but this induced hypogonadism. The patient was then placed on 30 mg/day of bromocriptine and 2 mg/day of haloperidol, and prolactin levels went down to the 50–80 μg/L range. The patient stopped taking haloperidol, and psychiatric symptoms returned. Zuclopenthixol was started at 4 mg/day, but prolactin levels were still elevated, so the patient was switched to 50 mg/day of clozapine, which lowered prolactin levels to below 30 μg/L.
Höfer et al. [28]	Acute psychosis	N/A	Amisulpride, olanzapine	1st: 185 ng/mL; 2nd: 70 ng/mL (62.2% decrease)	Initial size: 7 mm; final size: unchanged	N	N	The patient was a 52-year-old woman with a detected prolactinoma with acoustic and optic hallucinations, as well as restlessness, depression, and decreased libido. The patient had been taking 400 mg/day of amisulpride for 10 months. The patient was switched to 10 mg/day of olanzapine, and prolactin decreased significantly from 185 ng/mL to 70 ng/mL.
Maas et al. [29]	Paranoid schizophrenia	N/A	Thioridazine, valproic acid, thiothiexene, clozapine	1st: 76–135 μg/L; 2nd: 14 μg/L (86.7% decrease); 3rd: 6.3–10.5 μg/L (40.0% decrease)	Initial size; normal; 2nd scan: enlargement of the right side, suggestive of microadenoma; 3rd scan: normal following discontinuation of thioridazine; final size: normal.	N	N	The patient was a 19-year-old woman with a history of schizophrenia taking thioridazine and valproic acid (dosages unspecified). Thioridazine was discontinued and prolactin was measured 2 weeks later. Valproic Acid was used as a monotherapy for some time, but psychiatric symptoms recurred, so it was replaced by clozapine and electroconvulsive therapy. The patient was switched to thiothixene but had a recurrence of hyperprolactinemia and other similar symptoms, so clozapine therapy was reinstated.
Robbins et al. [30]	Paranoid schizophrenia	Bromocriptine	Chlorpromazine thioridazine	1st: 7,981 ng/mL; 2nd: 400 ng/mL (95.0% decrease); 3rd: 1,000 ng/mL (150% increase); 4th: 2,000 ng/mL (100% increase)	Initial size: not reported, described as large; 2nd measurement: no change; 3rd measurement: no change; 4th measurement: 10-15% change in the size of the tumor	Y	N	The patient was a 40-year-old with a 10-year history of schizophrenia, as well as chlorpromazine and thioridazine prescriptions (dosages unspecified). Following a diagnosis of prolactinoma, all previous antipsychotic medications were discontinued and bromocriptine was started. Thioridazine (25 mg/day) was restarted due to worsening psychiatric symptoms. Due to an increase in prolactin, thioridazine was discontinued and diazepam was started instead. This was not sufficient, and thioridazine (200 mg/day) was started once more and discontinued a bit later.
Weingarten and Thompson [31]	Paranoid schizophrenia	Bromocriptine	Thioridazine, clonidine	1st: 7295 ng/mL; 2nd: 200-400 ng/mL range (95.9% decrease); 3rd: 1956 ng/mL (552% increase); 4th: 366 ng/mL (81.2% decrease); 5th: 100 ng/mL (72.7% decrease)	Size not reported	Y	N	The patient was a 42-year-old with a 15-year history of paranoid schizophrenia and a myriad of neuroleptics were used for control. At the time the patient was diagnosed with prolactinoma, he was taking 600 mg of thioridazine 600 mg, 400-500 mg of diphenylhydantoin, and 200 mg of phenobarbital daily. The patient’s tumor was minimally debulked via craniotomy. Thioridazine was discontinued, and the patient was started on 7.5 mg of bromocriptine daily to decrease tumor size and prolactin levels. The patient was also given diazepam to control anxiety. The patient eventually had a relapse of psychotic symptoms after discontinuing thioridazine. It was reinstated at a 200 mg/day dosage. Four months later, the patient’s prolactin levels increased significantly. Thioridazine was discontinued and prolactin levels went down significantly. The patient redeveloped psychotic features six weeks after discontinuation of thioridazine and was started on clonidine, which was gradually increased to 0.5 mg daily. The patient is currently managed on clonidine and diazepam, and prolactin levels have remained around 100 ng/mL.
Burback et al. [32]	Mania with psychotic features	Cabergoline	Aripiprazole	1st: 48.1 μg/L; 2nd: 26.3 μg/L (45.3% decrease); 3rd: 6.2 μg/L (76.4% decrease); 4th: 11.4 μg/L (83.9% increase); 5th: 10.5 μg/L (7.89% decrease)	Initial size: 8 mm × 4 mm; 2nd measurement: 6 mm; final size: 6 mm	N	N	The patient was a 32-year-old woman who developed psychosis after being prescribed cabergoline. Cabergoline was discontinued and aripiprazole was started at 10 mg/day. Prolactin continued to decrease on this regimen. The aripiprazole dosage was decreased to 2 mg/day, and psychosis re-emerged, so it was increased back to 10 mg/day.
Pérez-Esparza et al. [33]	Acute psychosis	Cabergoline	Clozapine	1st: 13,494 ng/mL; 2nd: 5934.9 ng/mL (56.0% decrease); 3rd: 95.7 ng/mL (98.4% decrease)	Initial size: 46 mm × 27 mm × 37 mm; final size: “decreased significantly”	N	N	The patient was a 31-year-old woman with a prolactinoma who was started on 0.25 mg/week of cabergoline and developed psychiatric symptoms 2 weeks into treatment. Clozapine was started at 12.5 mg/day and titrated up to 50 mg/day The combination of 0.5 mg/week of cabergoline and 50 mg/day of clozapine brought the prolactin down to 95.7 ng/mL.
Casulari et al. [34]	Acute psychosis	Cabergoline	Quetiapine	1st: 14,992 ng/mL; 2nd: 1717 ng/mL (88.5% decrease); 3rd: 840 ng/mL (51.1% decrease); 4th: 646 ng/mL (23.1% decrease); 5th: 1049 ng/mL (62.4% increase)	Initial size: 4.0 cm × 2.5 cm; final size: not reported	Y	N	The patient was a 62-year-old man with an incidental prolactinoma discovered during imaging for a subdural hematoma. The patient had used cabergoline for ~17 years, with doses ranging from 1.0 to 3.5 mg/week. The patient developed psychiatric symptoms, which required combining the dosage of cabergoline (1.5 mg/week) with quetiapine (100 mg/day to 300 mg/day, and ultimately to 200 mg/day) and mirtazapine (30 mg/day). This regimen caused a decrease in the size of the prolactinoma, with a decrease seen in prolactin (see chart in Calsuri et al. [34]).

Control of Studies for Proper Comparison

Given the nature of this topic and the small number of case reports, it was difficult to control for individual confounders in each trial, such as dopamine agonist use, surgical interventions, and radiotherapy, and subsequently have enough data remaining for analysis. We also decided to describe each case in detail in Table 1 to allow readers to ascertain how differences in each case may have affected the outcomes of individual patients.

Control of Studies for Validity

To ensure validity, the study team decided to ensure that each report was conducted by physicians with expertise in psychiatry and endocrinology, and we deferred to provider wisdom, assuming that each patient was given a legitimate, standard form of care following certain guidelines and established norms.

Results

Our search yielded 27 studies, representing a total of 32 patients. From these patients, several themes arose regarding certain antipsychotics and the exacerbation of existing hyperprolactinemia and/or prolactinomas. More detailed descriptions of each patient can be found in Table 1.

Use of Risperidone

The first theme that arose was the use of risperidone and its association with hyperprolactinemia and subsequent prolactinoma formation. Risperidone was used in 17 of the reported patients [8-21]. Of these 17 patients, 13 were instructed by their physician to cease taking risperidone, after which prolactin levels decreased by an average of 66% [8,12-21]. In patients where risperidone was added or reinstated, prolactin levels increased by values ranging from 7.78% to 1910% [9,16,20].

Two patients had used risperidone in the past without complications. They had no clinical signs of hyperprolactinemia or prolactinoma on imaging until the use of another antipsychotic medication [11].

The two remaining patients who used risperidone were given a low dose in conjunction with cabergoline [19] or put on long-acting risperidone [9]. In the former, the patient developed galactorrhea and other signs of hyperprolactinemia six months after starting risperidone at a dosage of 3 mg/day. At the time of presentation, the patient’s prolactin level was 137 µg/L, and imaging revealed a 10 mm × 12 mm × 15 mm mass in the pituitary gland. Two years of cabergoline therapy (alongside risperidone) with a dosage of 0.5 mg thrice per week caused her serum prolactin levels to decrease to 8 µg/L. The size of the prolactinoma decreased by almost 7% to 12 mm × 10 mm × 14 mm. The patient was kept on 1 mg/day of risperidone with no reported issues.

The other patient [9] had an episode of psychosis, which was successfully treated with olanzapine. Afterward, the patient received an MRI, which revealed a pituitary macroadenoma measuring 1.6 cm in size and a prolactin level of 1986.5 ng/ml. The patient subsequently had surgical intervention, and prolactin levels fell to 291.8 ng/mL, which represented an 85.3% decrease. The patient was then put on long-acting risperidone to prevent subsequent psychosis, given the patient’s lack of compliance in the past. There was no psychological deterioration reported afterward, though there was a small increase in overall prolactin levels to 314.5 ng/mL. This only amounted to a 7.78% increase. The size of the adenoma did not change during the study period.

Because imaging findings were not always reported, it is difficult to ascertain the effect that risperidone discontinuation had on prolactinoma size. Additionally, surgical treatment in certain patients confounded the effects that decreasing dosage or discontinuation of the antipsychotic medications may have had on prolactinoma size.

Use of Haloperidol

Haloperidol was used in nine of the 32 patients reported [11,12,20,22-26]. Of these, three were instructed to discontinue their prescription, which was associated with an average decrease of 82% in prolactin levels [12,20,22].

One patient discontinued haloperidol but was reinstated on it, and another was non-compliant with haloperidol [27]. In the former, the patient had been taken off bromocriptine and haloperidol but was later reinstated on quinagolide (up to 0.375 mg/day) and haloperidol (1 mg twice per day). This had a net effect of decreasing the prolactin level by 91.6%. It is unknown how the addition of quinagolide and the reinstatement of haloperidol affected prolactin individually. In the latter patient, there was no recorded impact of non-compliance with haloperidol. Another patient utilized a small dose (3 mg/day) of haloperidol with 7.5 mg/day of bromocriptine to successfully treat both hyperprolactinemia and psychiatric symptoms [26]. A patient reported by Daradkeh et al. was discharged on 15 mg/day of haloperidol and 6 mg/day of benzhexol (an antimuscarinic muscle relaxant), but there was no measurement of prolactin after this treatment [24].

The final two patients had a history of haloperidol use. One had no signs of hyperprolactinemia on initial imaging; it only appeared after the use of a different drug (namely, amisulpride) [10]. The patient had previously been on haloperidol and biperiden but had been drug-naive for seven years. No information on dosage or length of use was given.

The other patient had been treated with a variety of medications (metofenazate, haloperidol, biperiden, fluphenazine decanoate, clozapine, and lithium) before being referred to the authors of the study [23]. It was suspected that this extensive history may have led to the conditions for hyperprolactinemia, given the antidopaminergic effects of antipsychotic medications. This antidopaminergic effect could have led to lactotroph hyperplasia, resulting in a prolactinoma sized at 16 mm × 19 mm and a baseline prolactin level over 1000 ng/mL.

Treatment was pursued using bromocriptine, which was discontinued during psychotic episodes and slowly reinstated afterward. Haloperidol, clozapine, diazepam, and perazine dimalonate were used to treat psychiatric episodes. Doses of haloperidol and clozapine were increased during episodes. The original doses were 150 mg/day of clozapine and 27 mg/day of haloperidol. It is unknown what the dose was increased to during episodes and what effect this had on prolactin levels. Pursuing treatment with 18.75 mg/day of bromocriptine for four years (except for during psychiatric episodes) led to a decrease in prolactin levels to 500 ng/mL (>50% decrease). Bromocriptine doses were maintained between 10 and 12.5 mg after that, and prolactin levels stabilized at 130 ng/mL (74% decrease) after an unspecified amount of time. The therapy described led to a decrease in the size of the tumor to a diameter of 8 mm.

Use of Amisulpride

Amisulpride was used in five of the 32 patients reported [11,20,28]. In the first three patients, amisulpride usage was associated with a significant increase in prolactin levels and resulted in newly formed foci, likely representing microadenomas on imaging [11]. These patients were verified as drug-naive for a baseline period prior to treatment and were then started on amisulpride at a dosage of 200 mg/day for 14 days. During the initial two-week time period, prolactin levels increased by an average of 644% in these three patients. In patient 1, there was an increase from 21.7 ng/mL to 109.10 ng/mL (402.8% increase). In patient 2, there was an increase from 18.55 ng/mL to 65.8 ng/mL (254.7% increase). Lastly, in patient 3, there was an increase from 6.7 ng/mL to 367.0 ng/mL (1274.5% increase).

After this initial two-week period, the dosage was increased to 800 mg/day, and prolactin levels were measured up to six months later. This increase in amisulpride dosage was associated with an increase in prolactin levels in all patients; however, the increase was not as severe as it was in the initial two-week period. Patient 1 experienced an increase from 109.10 ng/mL to 124 ng/mL (13.6% increase), patient 2 went from 65.8 ng/mL to 72.9 ng/mL (10.8% increase), and patient 3 went from 367.0 ng/mL to 424.0 ng/mL (15.53% increase). This only represented an average percent increase of 13.3%. Each patient experienced a slight decrease in prolactin levels by six months, although none of the levels were considered normal (shown in Table 1). There was no change in dosage, and patients 1 and 3 were only on biperiden for control of extrapyramidal symptoms. The initial imaging of all patients showed no abnormalities. After six months, all patients were re-imaged. Patient 1 exhibited two foci measuring 2 and 3 mm on the left and right superior parts of the gland, while patient 2 had a 2 mm foci on the left side of the pituitary gland, and patient 3 exhibited a 2 mm microadenoma in the left part of the hypophyseal gland.

In the latter two studies mentioned, amisulpride was discontinued for other medications. In one, it was discontinued for olanzapine [28]. Amisulpride had originally been prescribed at 400 mg/day for 10 months. This had led to a prolactin level of 185 ng/mL. A pituitary adenoma had been detected two months prior to admission. Imaging at the time of the visit showed a 7-mm hypointensity on the left side of the pituitary gland as well as a suspected microadenoma. Subsequent discontinuation of amisulpride for olanzapine (which was initially dosed at 10 mg/day) led to a decrease in prolactin to 70 ng/mL. This represented a 66.2% decrease in prolactin levels. After three months of olanzapine treatment (dosage unreported), the imaging was no longer able to confirm the 7-mm hypointensity, and the microadenoma had shrunk to a minuscule size.

In the other study, amisulpride was discontinued and replaced by a variety of other antipsychotics such as quetiapine, risperidone, haloperidol, and finally, olanzapine [20]. The patient originally received 300 mg of amisulpride per day after abruptly discontinuing biperiden (the patient had been on fluoxetine and alprazolam) and having headaches, visual disturbances, pseudoparkinsonism, and psychotic symptoms in the presence of normal cranial imaging. However, after about three to four months of amisulpride treatment, the patient reported endocrine symptoms and a PRL of 101 ng/mL. An MRI showed a 5 mm microadenoma without a corresponding mass effect. The patient was switched to 100 mg/day of quetiapine, and this led to a decrease in prolactin levels to 22.4 ng/mL (a 77.8% decrease). The effects of the other antipsychotic medications are reported in their respective sections.

Use of Thioridazine

Thioridazine was used in seven of the 32 patients presented [11,14,19,24,29-31]. Of these, three were instructed to discontinue their prescriptions, which was associated with an average decrease in the levels of prolactin by at least 89.7% [29-31]. Reinstating thioridazine in certain studies led to an average increase of 261% in prolactin levels [30,31].

Of the remaining four, one was given thioridazine previously and had no signs of hyperprolactinemia on initial imaging; it only appeared after the extensive use of amisulpride detailed earlier in this paper [11]. The patient had been off thioridazine and all antipsychotic medications for at least 20 days before treatment. Another patient had a history of thioridazine prescriptions, which were suspected of aiding prolactinoma formation [24]. The patient developed prolactinoma while using 150 mg/day of thioridazine alongside 15 mg/day of bromocriptine; symptoms did not subside until surgical resection and switching from thioridazine to 15 mg/day of haloperidol and 6 mg/day of benzhexol [24]. The final patient had used thioridazine in the past but experienced tardive dyskinesia, so she transitioned to risperidone and took 2 mg/day for 10 years before signs of prolactinoma [14].

Use of Aripiprazole

Aripiprazole was used in nine of the patients presented [8,10,11,15-17,21,25,32]. Of these patients, only two were instructed to discontinue treatment [8,16]. The former discontinued treatment since psychiatric symptoms were not adequately controlled [8]. The patient ultimately received transsphenoidal surgery, along with an unreported dosage of quetiapine, cabergoline, and levothyroxine [8]. Prolactin levels decreased to 52 ng/mL after surgery [8]. The latter patient had to discontinue treatment due to akathisia [16]. According to the authors, the patient was on 5 mg/day of aripiprazole and stopped after two months. This did not have a reported impact on prolactin levels or imaging findings. 

In the other studies [10,11,15,17,21,25,32], patients were switched to aripiprazole, with significant impacts on prolactin levels. The average decrease in prolactin levels after switching to aripiprazole from another antipsychotic was 67.65%.

Use of Olanzapine

Olanzapine was used in ten of the patients presented [9-13,16,18,20,28]. Of these, five were switched to olanzapine, significantly impacting prolactin levels [12,13,18,20,28]. The average decrease in prolactin levels after switching to olanzapine from other antipsychotics was 54.16%.

The other five patients [9-11,16] had a history of olanzapine use. Two patients had used olanzapine previously but had no signs of prolactinoma on initial imaging; findings that suggested microadenoma appeared after the use of amisulpride, which is detailed in an earlier section of this paper [11]. Another patient had previously used 10 mg/day of olanzapine to successfully treat a psychiatric episode [9]. Due to it being the first episode of psychosis, an MRI was performed, which found a 1.6 cm macroadenoma and a prolactin level of 1,986.5 ng/mL. Treatment was pursued through surgery and long-acting risperidone [9]. A patient reported by Freeman et al. had a four-year history of risperidone, olanzapine, and ziprasidone use. A macroadenoma was identified, which led to risperidone cessation [10]. Treatment was ultimately pursued via cabergoline cessation alongside intramuscular ziprasidone and lorazepam, which were ultimately switched to aripiprazole. Finally, Gupta et al. described a patient who had been using 0.5 mg/week of cabergoline and 10 mg/day of olanzapine but was switched to 5 mg/day of aripiprazole, which conferred a 94.7% decrease in prolactin levels [16]. 

Use of Clozapine

Clozapine was used in four of the patients presented [23,27,29,33]. Three of these patients were switched onto clozapine, which had a significant impact on prolactin levels [27,29,33]. The average decrease in prolactin levels after switching to clozapine from another antipsychotic was 68%.

One patient reported by Konopelska et al. had a history of using clozapine, along with many other antipsychotic agents [23]. A macroadenoma that was 16 mm × 19 mm in size was discovered, as were other antipsychotic agents. A macroadenoma that was 16 mm × 19 mm in size was discovered, and prolactin levels measured around 1000 ng/mL. The patient was prescribed 18.75 mg/day of bromocriptine, which was discontinued during psychiatric episodes but reinstated afterward. During psychiatric episodes, the patient was given increased doses of haloperidol and clozapine. The base doses were reported at 150 mg/day of clozapine and 27 mg/day of oral haloperidol. It is unknown what the doses were increased to. After four years of this treatment, prolactin levels were finally able to be reduced to 500 ng/mL and, eventually, 130 ng/mL.

Other Patients

There was another patient who did not fit into the themes detailed earlier but was included in our analysis. The patient had a prolactinoma discovered during imaging for a subdural hematoma. This patient had been using cabergoline for ~17 years, with doses ranging from 1.0 to 3.5 mg/week. This patient later developed psychiatric symptoms, which required combining the dosage of cabergoline (1.5 mg/week) with quetiapine (100 mg/day to 300 mg/day, and ultimately to 200 mg/day) and mirtazapine (30 mg/day). This regiment caused a decrease in the size of the prolactinoma, with a decrease seen in prolactin from 14,992 ng/mL to 1049 ng/mL [34].

Discussion

The recurring theme found in this analysis was that abnormally high levels of prolactin will decrease upon cessation of certain antipsychotic medications prescribed for schizophrenia and other psychotic diagnoses. Risperidone, amisulpride, and first-generation antipsychotics (FGAs) such as thioridazine, thiothixene, and haloperidol have been reported to have a high prevalence of hyperprolactinemia and an overall prolonged rise in prolactin levels. Risperidone and most FGAs have high binding affinities for slow dissociation from the D2 receptor [35]. The D2 receptor in pituitary lactotrophs is typically occupied by dopamine, whose binding exerts an inhibitory effect on prolactin release. Thus, the higher binding affinity of certain antipsychotics decreases the opportunity for inhibitory dopamine to bind pituitary lactotroph cells, ultimately leading to increased levels of prolactin. However, amisulpride and haloperidol also cause elevations of PRL levels but show relatively fast dissociation profiles. This suggests that factors other than kinetics affect the prolactin-releasing properties of antipsychotics, such as their ability to cross the blood-brain barrier (BBB), which will be discussed later in this section [36]. Additionally, some second-generation antipsychotics (SGAs), such as clozapine, amisulpride, risperidone, and olanzapine, have been found to be more efficacious than FGAs despite having lower binding 4 affinity for the D2 receptor [37]. SGAs are sometimes recommended over FGAs due to the decreased chance of adverse effects; this study also found that certain SGAs, namely risperidone and olanzapine, were also superior to FGAs in treating certain positive and negative symptoms of schizophrenia, as well as some symptoms that are untouched by FGAs. One study utilized the Positive and Negative Syndrome Scale (PANSS) to compare the efficacy of risperidone versus haloperidol. It was found that risperidone was better at improving the positive symptoms of suspiciousness and various negative symptoms that were unaffected by haloperidol, such as blunted affect, emotional withdrawal, and passive-apathetic social withdrawal. Other symptoms improved by risperidone but untouched by haloperidol included grandiosity, depression, and disturbances of volition, among others [38]. Thus, it is possible that, in some cases, SGAs should be considered first-line. Furthermore, more studies should be conducted to determine what properties of these SGAs make them better for certain symptoms.

Certain predisposing factors, such as having genetic D2 receptor polymorphisms, being a woman of reproductive age, or being an adolescent, can also explain why some people are more at risk of developing hyperprolactinemia [35]. Additionally, increases in estrogen, thyrotropin-releasing hormone (TRH), angiotensin II, antidiuretic hormone (ADH), and other substances as a result of disease or as a side effect of antipsychotic use can also increase the secretion of prolactin [36]. It is possible that some antipsychotics increase levels of these substances and subsequently cause increased secretion of prolactin. For example, certain antipsychotics such as haloperidol have been associated with the syndrome of inappropriate ADH (SIADH), which is characterized by increased ADH secretion. It is also proposed that SIADH is partly caused by increased stimulation of central serotonin receptors, which also stimulates prolactin release [39]. Increased stimulation of central serotonin receptors via drugs that increase extracellular serotonin concentrations or act as direct agonists to the 5-hydroxytryptamine (5-HT) receptor has been suggested as another mechanism through which antipsychotics may cause increased secretion of prolactin [36]. It is suggested that 5-HT indirectly stimulates prolactin secretion through a complex pathway involving both the hypothalamus and pituitary. One antipsychotic medication that may act through this serotonergic pathway is risperidone, which, despite acting as a 5-HT receptor antagonist, increases extracellular serotonin levels [40]. The possibility of other mechanisms through which antipsychotics can increase prolactin levels should be explored in order to develop more potential drug targets.

The permeability of the blood-brain barrier (BBB) to certain medications can explain why some patients given thioridazine or haloperidol did not develop a prolactinoma initially but did develop a prolactinoma after being given another drug such as amisulpride. The pituitary gland and therefore the D2 receptors of its secretory lactotroph cells are located outside of the blood-brain barrier. Thus, amisulpride, which has a decreased ability to cross the BBB, may cause prolonged interaction with the D2 receptors and less opportunity for inhibitory dopamine to bind, resulting in increased prolactin release [36]. Additionally, due to their decreased membrane permeability, amisulpride and risperidone have both been reported to have a higher ratio of pituitary to striatal D2 receptor occupancy as compared to other second-generation antipsychotics [35]. Thus, higher concentrations of these drugs may be required to have the same effect on the striatal receptors where the antipsychotic effects are intended to occur. This could explain why patients using long-acting injectable forms of risperidone, which result in a lower average steady-state plasma concentration than oral risperidone, did not show significant exacerbations of prolactin levels [41]. Another consideration to be made is regarding the active drug metabolites; one study suggests the 9-hydroxy metabolite of risperidone is less able to cross the BBB and has been found to cause increased prolactin concentrations when risperidone did not [35]. This study also suggests that those with more rapid CPY2D6 metabolism tend to have increased concentrations of 9-hydroxyrisperidone; thus, it would be helpful to consider the implications of genetic cytochrome P450 polymorphisms on the accumulation of active drug metabolites and the effects of these metabolites on prolactin levels. Further studies on the membrane permeability and metabolite profiles of drugs can aid in developing treatment options with fewer side effects.

Due to the clinical complications that arise upon the development of hyperprolactinemia, it is important to explore treatment options for schizophrenia that would not induce this issue. In this analysis, patients who switched from antipsychotics such as amisulpride onto olanzapine showed an overall decrease in prolactin levels, including one patient who showed a decrease in prolactinoma size. This is consistent with another study that has shown olanzapine to show lesser elevations in prolactin levels compared to other antipsychotics such as haloperidol and risperidone [42]. This study suggests that newer atypical antipsychotics such as olanzapine and clozapine likely show less elevation in prolactin levels due to lower D2-binding affinities; as mentioned before, antagonists with high binding affinity to D2 receptors would cause decreased binding of inhibitory dopamine and increased prolactin levels. In the cases analyzed here, patients who were switched from other antipsychotics to clozapine were also found to show decreased elevations in prolactin levels, including one patient who displayed a significant decrease in prolactinoma size while on clozapine and cabergoline. However, clozapine is not a first-line treatment due to significant side effects such as agranulocytosis, myocarditis, metabolic syndrome, and seizures, among others. These side effects impart the need for close monitoring, such as weekly blood tests, further raising the issue of patient non-compliance [43].

A promising option for treating schizophrenia that confers a low risk of developing hyperprolactinemia is aripiprazole, which is a partial agonist for the D2 receptor [44]. One patient in this study who was initially taking olanzapine and cabergoline showed a significant decrease in prolactin levels upon starting aripiprazole. As aripiprazole is a partial dopamine agonist, it has both antagonistic and agonistic properties; in areas of hyperdopaminergic activity, it acts as an antagonist, and in areas of hypodopaminergic activity, it acts as an agonist [45]. Thus, aripiprazole does not cause a complete blockade of dopaminergic receptors, as is the case with most antipsychotics used to treat schizophrenia. Since there is no complete antagonism of dopamine receptors, there is still dopamine available to inhibit excessive prolactin release, lowering the risk of developing hyperprolactinemia. However, if a patient’s condition is not stabilized by aripiprazole, it is more important to consider the patient’s psychiatric stability as opposed to the risk of hyperprolactinemia. The main medications used to maintain normal prolactin levels are dopamine agonists, such as cabergoline and bromocriptine, which may not be the ideal choice for patients with a history of psychosis as they can increase the risk of psychiatric decompensation. Bromocriptine is associated with side effects such as nausea, vomiting, orthostatic hypotension, headaches, mood elevation, and pathological gambling. Both cabergoline and bromocriptine may cause nightmares, hallucinations, psychosis, and insomnia. Thus, in these patients with schizophrenia or other mood disorders, surgical resection of the prolactinoma can be considered, but monitoring of prolactin levels and closer surveillance of the prolactinoma may be the best option [9]. Still, while bromocriptine-induced new-onset psychosis has many examples in the literature, using cabergoline in conjunction with the originally prescribed antipsychotic has rarely been found to exacerbate the underlying psychiatric issue [44]. This coincides with the findings in this analysis, as there were patients whose hyperprolactinemia and psychiatric symptoms were both successfully treated through the use of haloperidol, olanzapine, or clozapine with bromocriptine and risperidone or clozapine with cabergoline; these patients also showed decreases in prolactinoma size on imaging.

This analysis was limited by the relatively small sample size and limiting searches to English only. Additionally, due to a lack of imaging findings, a lack of consistent reporting of prolactinoma size, and some of the patients undergoing surgery, it was difficult to conclusively determine the effect of some antipsychotics on prolactinoma growth. Future studies can be done on antipsychotic characteristics such as membrane permeability, metabolite profiles, and off-target activity to fully understand the effect of antipsychotics on prolactinoma development, treatment, and symptom management.

## Conclusions

After analyzing the literature as a whole, it is evident that risperidone, haloperidol, thioridazine, and amisulpride impact prolactinoma growth, as cessation leads to a decrease in serum prolactin levels. Certain APs fared better than others in regard to prolactinoma growth, including clozapine, aripiprazole, and olanzapine. While prolactin levels showed clear trends in relation to certain antipsychotic medications, imaging findings were not nearly as conclusive. The infrequency of imaging made it difficult to ascertain the individual impact of certain antipsychotics on prolactinoma size. Also, there were often confounding factors, such as surgical and radiation treatments.

Ideally, providers should focus on balancing detrimental prolactinoma symptoms (lactation, infertility, etc.) while also focusing on a treatment course that explicitly addresses a patient's psychiatric health needs. This approach requires preferring certain APs over others, considering dopamine agonist therapy, or closer prolactinoma surveillance.
